# Territory Quality and Plumage Morph Predict Offspring Sex Ratio Variation in a Raptor

**DOI:** 10.1371/journal.pone.0138295

**Published:** 2015-10-07

**Authors:** Nayden Chakarov, Martina Pauli, Anna-Katharina Mueller, Astrid Potiek, Thomas Grünkorn, Cor Dijkstra, Oliver Krüger

**Affiliations:** 1 Department of Animal Behaviour, Bielefeld University, PO Box 10 01 31, 33501, Bielefeld, Germany; 2 BioConsult SH, Brinckmannstr. 31, 25813, Husum, Germany; 3 Behavioural Biology, University of Groningen, PO Box 11103, 9700 CC, Groningen, The Netherlands; University of Lausanne, SWITZERLAND

## Abstract

Parents may adapt their offspring sex ratio in response to their own phenotype and environmental conditions. The most significant causes for adaptive sex-ratio variation might express themselves as different distributions of fitness components between sexes along a given variable. Several causes for differential sex allocation in raptors with reversed sexual size dimorphism have been suggested. We search for correlates of fledgling sex in an extensive dataset on common buzzards *Buteo buteo*, a long-lived bird of prey. Larger female offspring could be more resource-demanding and starvation-prone and thus the costly sex. Prominent factors such as brood size and laying date did not predict nestling sex. Nonetheless, lifetime sex ratio (LSR, potentially indicative of individual sex allocation constraints) and overall nestling sex were explained by territory quality with more females being produced in better territories. Additionally, parental plumage morphs and the interaction of morph and prey abundance tended to explain LSR and nestling sex, indicating local adaptation of sex allocation However, in a limited census of nestling mortality, not females but males tended to die more frequently in prey-rich years. Also, although females could have potentially longer reproductive careers, a subset of our data encompassing full individual life histories showed that longevity and lifetime reproductive success were similarly distributed between the sexes. Thus, a basis for adaptive sex allocation in this population remains elusive. Overall, in common buzzards most major determinants of reproductive success appeared to have no effect on sex ratio but sex allocation may be adapted to local conditions in morph-specific patterns.

## Introduction

Parents should preferentially produce the sex with the highest fitness value under the prevailing environmental conditions [1–4]. One basic determinant of fitness is the difference in resource demands between offspring of both sexes and the resulting difference in infant mortality under harsh conditions [2, 5]. In consequence, parents may adapt their offspring sex ratio to the local conditions. The conjunction of the “costly sex” and “local adaptation” hypotheses implies that in harsh conditions, such as prey-deficient years and in poor-quality territories, parents should preferentially produce the less demanding sex [2, 6, 7]. Another basic fitness component is the length of reproductive careers (timespan in which individuals attempt to reproduce). Reproductive lifespan can differ between sexes and hence may explain sex-ratio variation [8]. In birds, being raised early in the breeding season may enable one sex to recruit earlier and have a longer reproductive career than the other sex, raised under the same conditions. In monogamous species, reproductive career length is strongly correlated with fitness, thus providing an incentive for sex allocation over the breeding season [8]. Such an allocation should lead to different distributions of reproductive career length between sexes. Under this “early sex hypothesis”, offspring of the more rewarding sex should be produced earlier in the season than the less rewarding sex. Finally, the lifetime reproductive success (LRS, here used as the number of fledglings produced over the individual lifetime) is a major component of fitness and its variation gives a good impression of fitness variance in a population, albeit it does not account for variance in pre-reproductive survival [9]. Although the overall mean LRS should be equal between the sexes, differences in the variance and distribution of LRS between sexes would present a cause for adaptive sex allocation.

In this study, we use a large dataset of reproductive performance and offspring sex of common buzzards *Buteo buteo*, encompassing the entire lifetime reproductive output of individuals. Common buzzards, like most birds of prey, exhibit reversed sexual size dimorphism (RSD), with female offspring fledging up to 20% heavier and therefore probably demanding greater parental investment than males [10–13]. Females of medium-sized birds of prey, such as marsh harriers *Circus aeruginosus* and goshawks *Accipiter gentilis*, may accelerate the start of their reproductive careers, if fledged early in the season [8], for similar patterns in ungulates see [14]. Similarly, some female buzzards could have longer breeding careers and if so, the variance and other aspects of the distribution of breeding career length may differ between sexes. An earlier study showed that female buzzards indeed may have a slightly longer reproductive lifespan and slightly more breeding attempts than males, without differences in mean LRS [15]. With a mean brood size of two and no sign of sex-biased dispersal [16, 17] in buzzards, the “local adaptation hypothesis” predicts increased male production under harsh conditions, while the “early sex hypothesis” predicts increased female production earlier in the breeding season [14].

Furthermore, common buzzards appear in three plumage morphs (dark, intermediate and light) which are inherited in a Mendelian fashion [18, 19]. Buzzard plumage morphs differ substantially in individual fitness (intermediates having higher LRS than extreme morphs; [15, 20]), behaviour (light males are more aggressive than intermediate and dark ones; [21]), immunity (intermediates have lower cellular and higher specific humoral response; Chakarov et al. unpublished manuscript) and parasite loads [endoparasite infection intensity decreases and ectoparasite infestation increases with melanisation; [20]. Each of these traits could influence sex allocation but due to their possible complex interactions we refrain from directed predictions about the specific sex allocation of morphs.

Here, we first search for correlates of sex ratio among variables known to influence sex allocation in other systems. Among them are: Laying date (corresponding to predictions of the “early sex hypothesis”) [8], prey availability [22, 23], and territory quality (corresponding to predictions of the “local adaptation hypothesis”) [24, 25], as well as parental plumage morph [26, 27] and parental age [28], being correlates of reproductive performance [15, 19]. In buzzards, plumage morph and prey availability are the two main drivers of reproductive investment, therefore their interaction may also be expected to affect sex allocation [15, 29–31]. We analyse two hierarchical levels of sex variation: (1) Nestling sex allows to test hypotheses about effects of individual offspring traits, such as laying date corresponding to the “early chick hypothesis”[32]. (2) Lifetime sex ratio provides us with the rare opportunity to search for effects of lifetime reproductive strategies on offspring sex ratios and to identify potential constraints of adaptive sex allocation such as genetically-encoded polymorphisms or maternal effects, although it is not a good parameter to study short-term adaptations of sex ratio. Finally, we test whether the three previously discussed premises for adaptive sex allocation (higheroffspring mortality corresponding to the “costly sex hypothesis”, longer reproductive lifespan, and higher lifetime reproductive success in either sex) are met in common buzzards.

## Materials and Methods

### Ethics statement

Blood samples were collected for common buzzard nestlings via venepuncture. All field studies and animal handling were performed with permission from the local authority Kreis Gütersloh, permit nr: 4.5.2-723-Bussard in accordance to German federal and state laws.

### Study site

The study was carried out in a ca. 300 km^2^ area in eastern Westphalia, Germany (8°25′ E and 52°06′ N) between 1989 and 2012 with sex ratio data being available from 2002–2012. The habitat consists of pastures and meadows, interspersed with woodlots, varying between 0.001 and 7 km^2^ in size [33, 34].

### Population and explanatory variables

Buzzard population studies with individual identification of breeding birds in this area have been performed since 1989 (e.g. [30, 35, 36]). Buzzards have distinctive individual plumage pigmentation patterns, allowing individual recognition without artificial marking. In more recent years, photographing individuals and genetic fingerprinting of buzzard chicks were also used to increase the resighting accuracy [37]. Buzzards, like other birds of prey, have a high breeding site fidelity throughout their lives [38]. For all breeding buzzards, the first breeding attempt in the study area was assumed to be the beginning of the reproductive career, and thus a minimum age is known. An individual was assumed to be dead if it was not recorded in the area for at least two consecutive years [9]. LRS for individuals with completely known breeding life-histories was calculated as the total number of fledglings produced during their lifetime. Partner change can happen between years, so LRS of female and male breeding partners can differ. Territory quality was defined as the proportion of years a territory was occupied since it was used for the first time [39] and is a good predictor of reproductive success [40]. Since our dataset spans more than several generations of territory holders, we consider environmental quality to be a substantial component of territory quality.

Common buzzards are highly territorial, specialised raptors, and density of voles, their main prey, strongly predicts annual fluctuations in buzzard reproductive output [23, 41]. To estimate food availability, we scored vole abundance at the beginning of the breeding season, using three categories: low, intermediate or high. We used the re-opened holes method, where the number of active holes per unit area is counted and no trapping is performed [42]. This score strongly predicts the fraction of common voles *Microtus arvalis* among all prey items found in the nest when chicks are sampled (r = 0.746, df = 9, P < 0.001). Since 2002, all accessible nests were climbed (85% of all successful broods) and nestlings ringed. Tarsus length was measured with a calliper to the nearest 0.1 mm, wing length with a ruler to the nearest 1 mm, and weight was taken with a Pesola spring balance to the nearest 5 g. Age of the chicks was estimated by comparison of morphometric measurements with a standard, sex-specific growth curve [43]; mean age at ringing 29 ± 6 days] and subtracted from day of the year in order to derive hatching and laying date estimates, assuming an incubation period of 34 days (Mebs 1964). After ringing the nest was visited short before the estimated day of fledging to count the number of visible fledglings. The number of fledglings of the brood is further referred to as brood size. During the breeding season, morph of the female and male territory holders (further referred to as mother and father) and of the nestlings were recorded. Individuals with dark head, heavily speckled or dark breast and underwing coverts were considered as dark. Birds with dark head, intermediately speckled breast and underwing coverts were scored as intermediate. Birds with little or no melanisation of breast and underwing coverts, in extreme cases with light head and upperwing coverts were scored as light. From each ringed nestling, a blood sample of ca. 200 μl was taken via venipuncture of the brachial vein and used for DNA-sexing following a standard protocol [44]. More than 100 samples were scored more than once to verify repeatability of molecular sexing. DNA-sexing results were compared with morphological sexing, common in size-dimorphic birds of prey [45]. No inconsistencies were detected between both methods.

### Datasets and statistical analyses

Overall, 1780 nestlings from 881 broods were sexed. This included 43 chicks which died before fledging and were used to test for sex-specific mortality before fledging. To explain nestling sex ratio in the final dataset, we used binomial generalized linear mixed models with logit link. Factors used to explain nestling sex were the morphs of both parents and of the nestling, and annual vole score. The age of both parents (i.e. current length of their reproductive career), date of laying, brood size and territory quality were entered as covariates. The final dataset with data available for all variables contained 1678 nestlings. Brood and mother identities were entered as nested random factors in order to account for potential non-independence of sibling sexes. Lifetime sex ratio was computed as the proportion of males among hatchlings produced during the lifetime of a parent. Individual-specific explanatory variables were lifetime reproductive success (LRS), the individual’s and partner’s melanin morphs (in case of multiple partners the mean morph of the partners was computed with light and dark considered as extremes and intermediates as being 50% of both extremes), the length of the individual reproductive career (longevity) and territory quality, after assuring that included individuals had not switched territories.

LRS, observed longevity (corresponding to the length of the reproductive career in our data) and lifetime sex ratio were analysed for 109 mothers of 432 nestlings and 94 fathers of 384 nestlings with fully known life histories and where all offspring were sexed. These datasets include only individuals which have started breeding after 2001 and therefore have no overlap with a sample from the same population used for previous LRS analyses [15]. Lifetime sex ratio was analysed with GLMMs with Gaussian distribution with cohort of the individual included as a random term. A complete description of all datasets and models is included in Tables 1–5.

**Table 1:** Datasets for analysis of nestlings sex, lifetime sex ratio of females and males, lifetime reproductive success, reproductive lifespan (longevity) and mortality of nestlings.

**(A) Dataset for analyses of nestling sex (n = 1678)**	
**Dependent variable**	**Type of data and coding**
Nestlings sex	Binomial (0- female, 1-male)
**Explanatory variables**	
Date of laying	Day since 1. June (3–57, covariate)
Size hierarchy in the brood	Size rank of chick in nest (1–4, covariate)
Brood size	Number of siblings in brood (1–4, covariate)
Territory quality	Proportion of years the territory was occupied after its establishment between 1989–2012 (0.04–1, covariate)
Minimum age of mother	Years (2–18, covariate)
Minimum age of father	Years (2–18, covariate)
Plumage morph of nestling	Dark, intermediate or light (factor)
Plumage morph of mother	Dark, intermediate or light (factor)
Plumage morph of father	Dark, intermediate or light (factor)
Vole score	High, intermediate, low (factor)
Year	Year of hatching, 2002–2012 (factor)
**(B) Dataset for analyses of lifetime sex ratio of females (n = 109)**	
**Dependent variable**	**Type of data and coding**
Lifetime sex ratio	Ratio of males produced by the individual (0–1, covariate)
**Explanatory variables**	
Territory quality	Proportion of years the territory was occupied after its establishment between 1989–2012 (0.04–1)
Lifetime reproductive success (LRS)	Total number of fledglings produced by the individual over its entire lifetime
Longevity	Minimum age of the individual when registered as dead (2–18)
Plumage morph	Dark, intermediate or light
Plumage morph of male partner	Dark, intermediate or light
Cohort	Year in which the individual first bred
**(C) Dataset for analyses of lifetime sex ratio of males (n = 94)**	
**Dependent variable**	**Type of data and coding**
Lifetime sex ratio	Ratio of males produced by the individual (0–1, covariate)
**Explanatory variables**	
Territory quality	Proportion of years the territory was occupied after its establishment between 1989–2012 (0.04–1)
Longevity	Minimum age of the individual when registered as dead (2–18)
Plumage morph	Dark, intermediate or light
Cohort	Year in which the individual first bred
**(D) Dataset for analyses of lifetime reproductive success and length of reproductive career (i.e. minimum age of individuals not found the population > 2 years), n = 205, 109 females, 94 males**	
**Dependent variables**	**Type of data and coding**
Lifetime reproductive success	Total number of fledglings produced by the individual over its entire lifetime
Longevity	Minimum age of the individual when registered as dead (2–18)
**Explanatory variables**	
Sex of adult	Binomial (0- female, 1-male)
**(E) Dataset for analyses of nestling mortality (n = 1780)**	
**Dependent variables**	**Type of data and coding**
Nestling survival	Nestling does not fledge (0) or fledges (1)
**Explanatory variables**	
Sex of nestling	Binomial (0- female, 1-male)
Voles score of year	High, intermediate, low
Territory quality	Proportion of years the territory was occupied after its establishment between 1989–2012 (0.04–1, covariate)

**Table 2:** Initial (A) and best (B) models of nestlings sex in the dataset including all sampled common buzzard nestlings (n = 1678). ANOVA between initial and best model of nestling sex χ^2^ = 21.548, df = 21, P = 0.426, ΔAIC = 19.6.

**(A)**				
**Explanatory variable**	**Estimate**	**SE**	**z-value**	**P-value**
Intercept	1.04577	0.69841	1.497	0.1343
Territory quality	-0.88137	0.28307	-3.114	0.00185
Date of laying	0.11236	0.07708	1.458	0.1449
Size hierarchy in the brood	0.04787	0.0717	0.668	0.50438
Brood size	-0.00518	0.07615	-0.068	0.94576
Minimum age of mother	-0.07824	0.20851	-0.375	0.70748
Minimum age of father	0.19467	0.20076	0.97	0.33224
Plumage morph of nestling dark-intermediate	-0.12667	0.17045	-0.743	0.45739
dark-light	-0.20157	0.20716	-0.973	0.33054
Plumage morph of motherdark-intermediate	-0.75395	0.38953	-1.936	0.05293
dark-light	0.09288	0.43348	0.214	0.83034
Plumage morph of father dark-intermediate	-0.16455	0.47489	-0.346	0.72897
dark-light	-0.56501	0.50617	-1.116	0.26432
Vole score low-intermediate	-1.00993	0.77827	-1.298	0.19441
low-high	-0.70453	0.65285	-1.079	0.28052
Morph of father*Vole score intermediate:low	0.4117	0.66298	0.621	0.53461
light:low	0.6653	0.69704	0.954	0.33985
interm:intermediate	-0.01611	0.54168	-0.03	0.97628
light:intermediate	0.42169	0.57378	0.735	0.46238
Morph of mother*Vole score intermediate:low	0.92167	0.512	1.8	0.07184
light:low	0.10733	0.56187	0.191	0.84851
interm:intermediate	1.0122	0.4594	2.203	0.02757
light:intermediate	0.32522	0.49906	0.652	0.51461
**(B)**				
**Explanatory variable**	**Estimate**	**SE**	**z-value**	**P-value**
Intercept	0.5465	0.2050	2.666	0.00768
Territory quality	-0.7053	0.2678	-2.634	0.00845

**Table 3:** Initial (A) and best (B) models of lifetime sex ratio of female common buzzards with completely known life histories (n = 109). ANOVA between initial and best model of female lifetime sex ratio χ^2^ = 8.503, df = 6, P = 0.203, ΔAIC = 3.5.

**(A)**				
**Explanatory variable**	**Estimate**	**SE**	**t-value**	**P-value**
Intercept	0.803218	0.231904	3.464	0.000786
Territory quality	-0.06065	0.148115	-0.409	0.683091
LRS	-0.00949	0.016222	-0.585	0.55966
Individual longevity	-0.00731	0.025165	-0.29	0.772189
Plumage morph of female dark-intermediate	0.106185	0.099023	1.072	0.286233
dark-light	0.007444	0.110822	0.067	0.946584
Mean plumage morph of male partners	-0.06851	0.057839	-1.184	0.239094
**(B)**				
**Explanatory variable**	**Estimate**	**SE**	**t-value**	**P-value**
Intercept	0.54151	0.05335	10.15	<0.001

**Table 4:** Initial (A) and best (B) models of lifetime sex ratio of male common buzzards with completely known life histories (n = 94). ANOVA between initial and best model of female lifetime sex ratio χ^2^ = 0.943, df = 2, P = 0.624, ΔAIC = 3.1.

**(A)**				
**Explanatory variable**	**Estimate**	**SE**	**t-value**	**P-value**
Intercept	0.872276	0.15068	5.789	
Territory quality	-0.437929	0.164109	-2.669	0.011
LRS	0.004587	0.012739	0.36	0.719
Individual longevity	0.022321	0.033785	0.661	0.511
Plumage morph of male dark-intermediate	-0.101125	0.121425	-0.833	0.016
dark-light	-0.305691	0.130805	-2.337	
**(B)**				
**Explanatory variable**	**Estimate**	**SE**	**t-value**	**P-value**
Intercept	0.89087	0.14824	6.01	
Territory quality	-0.38509	0.1542	-2.497	0.017
Plumage morph of male dark-intermediate	-0.09077	0.1222	-0.743	0.023
dark-light	-0.27835	0.13082	-2.128	

**Table 5:** Generalized linear models of lifetime reproductive success, LRS (A), reproductive lifespan, longevity (B) and nestling mortality (C).

**(A)**				
**Explanatory variable**	**Estimate**	**SE**	**z-value**	**P-value**
Intercept	1.377078	0.048113	28.622	<0.001
Sex	-0.009217	0.070135	0.131	0.895
**(B)**				
**Explanatory variable**	**Estimate**	**SE**	**z-value**	**P-value**
Intercept	0.99902	0.05812	17.188	<0.001
Sex	0.02389	0.0844	0.283	0.777
**(C)**				
**Explanatory variable**	**Estimate**	**SE**	**z-value**	**P-value**
Intercept	3.2131	0.3855	8.335	<0.001
Sex (male compared to female)	0.3073	0.5954	0.516	0.6058
Vole score (intermediate compared to low)	0.9405	0.698	1.347	0.1778
Vole score (high compared to low)	1.1997	0.5633	2.13	0.0332
Male in intermediate vole year (compared to female in such)	0.7808	1.3028	0.599	0.549
Male in high vole year (compared to female in such)	-1.5127	0.7568	-1.999	0.0456

Maximal models contained all variables without missing values in the final dataset. Model selection was based on AIC model weights [46]. Statistical modelling was performed in R 3.1.1, using the packages lme4 1.1–7 and MuMIn 1.10.5 [47]. Plots of sex ratio against all independent variables were visually inspected for non-linear relationships. Values are given and plotted ± SE. All datasets, full initial and best final models are summarised in Tables 1–5.

## Results

### Nestling sex

**Fig 1:**
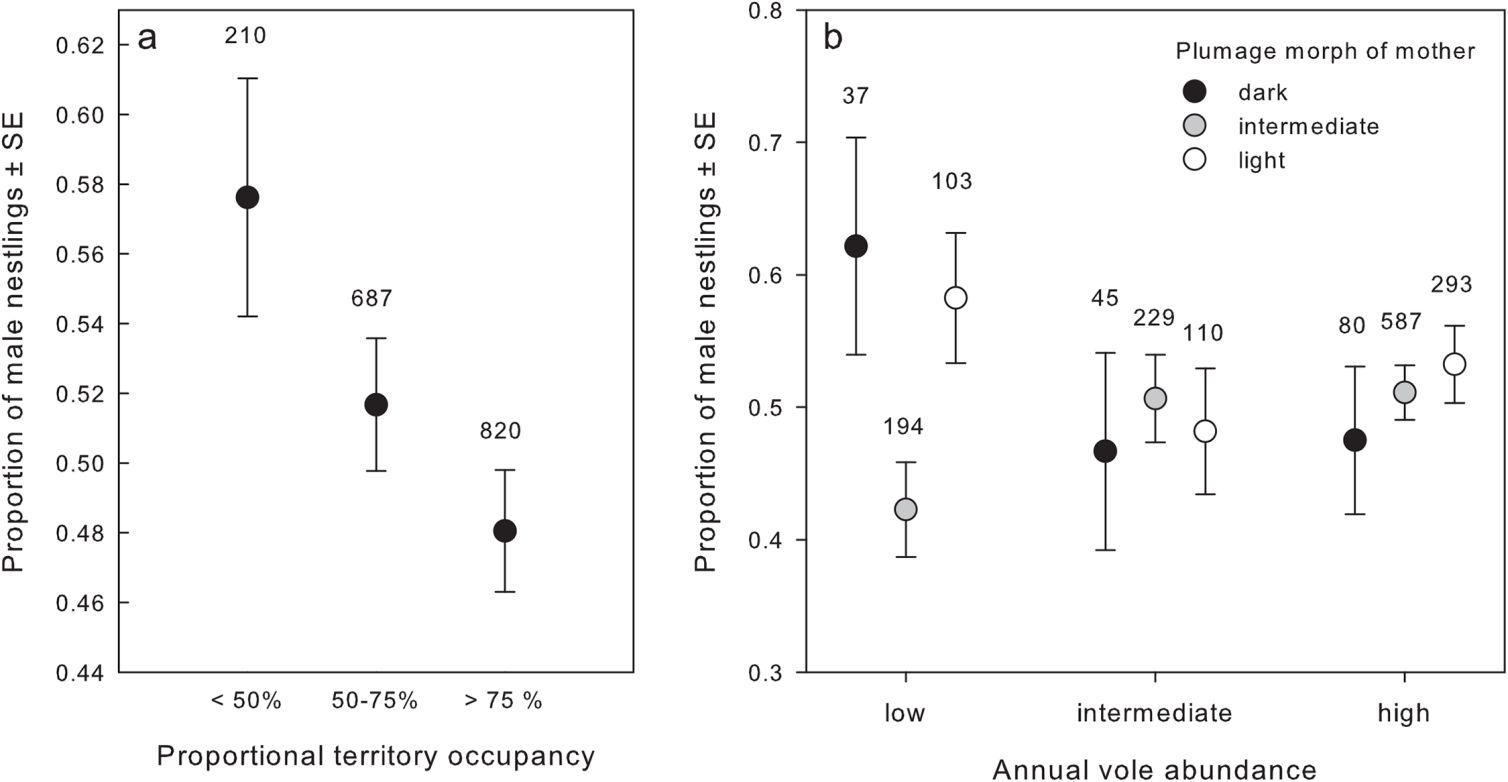
Sex ratio of nestling buzzards hatched (a) in poor, intermediate and good territories based on their proportional occupancy and (b) in years with low, intermediate and high vole abundance to dark intermediate and light mothers. Binning into three territory quality classes is for visual purposes only. Statistical analyses were performed with the continuous variable territory quality.

The overall sex ratio in our most comprehensive dataset did not significantly deviate from random (50.7% male, Neuhäuser test, z = 0.563, P = 0.645, N = 1780; test described in Neuhäuser 2004 [48]. The best model explaining nestling sex in the full dataset contained territory quality (Table 2). More female nestlings hatched in territories of better quality (Fig 1). Additionally, the interaction of annual vole score and plumage morph of the mother was a significant predictor of nestling sex ratio (χ^2^ = 9.552, df = 4, P = 0.049). In low vole years, both light and dark mothers produced more male nestlings, while intermediate mothers produced more female offspring (Fig 1), and there was no difference in nestling sex ratio in years of intermediate and high vole abundance. This interaction of maternal morph and vole score, however, was not included in the best model explaining nestling sex (ΔAIC = 1.6 between best model and model including vole score × morph of mother).

### Lifetime sex ratio

**Fig 2:**
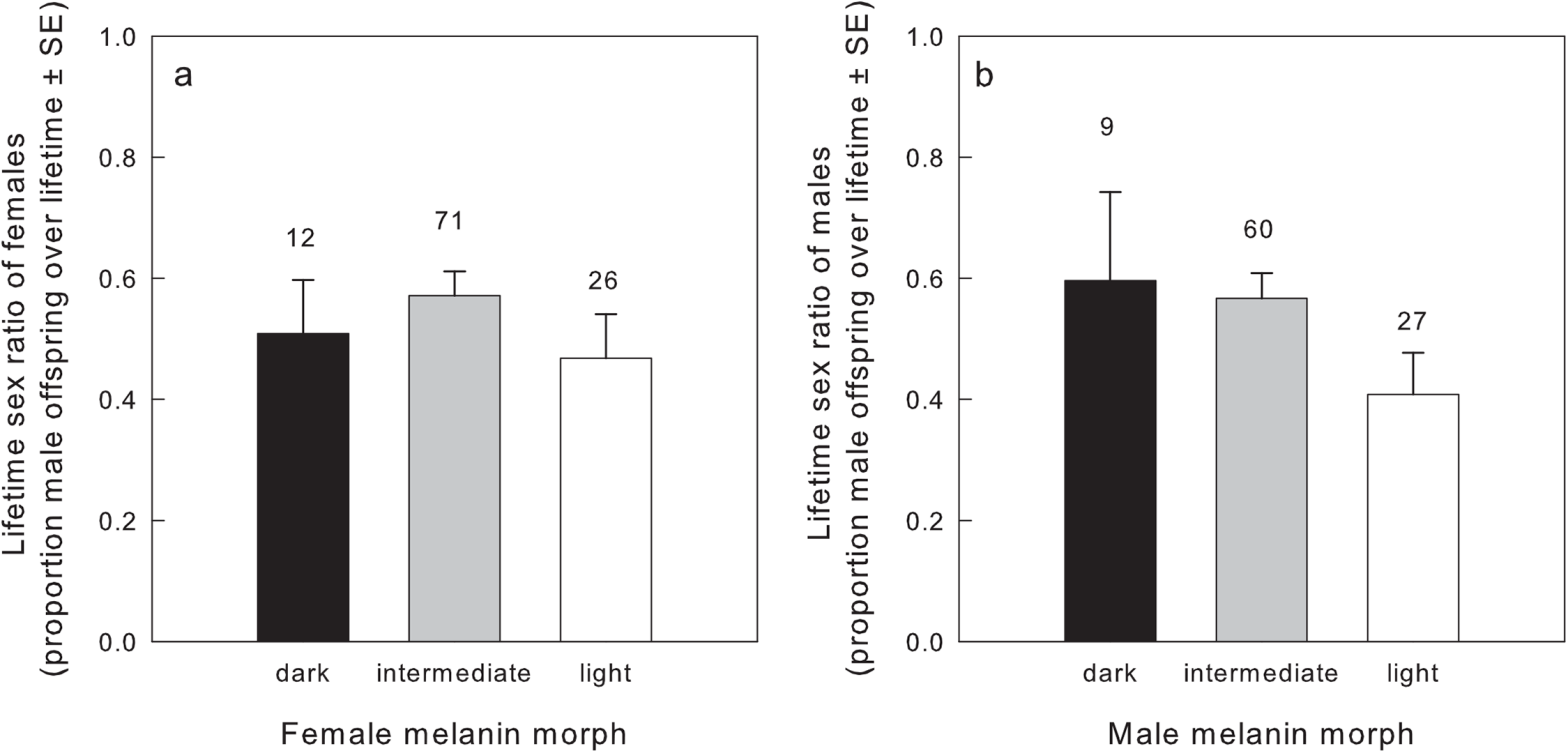
Lifetime sex ratio (+SE) of (a) mothers and (b) fathers of different melanin morphs with entirely known reproductive output. Sample sizes are number of individuals of the respective class with completely known lifetime sex ratio.

We found no significant predictors of female lifetime sex ratio (LRS: χ^2^ = 0.652, df = 1; longevity: χ^2^ < 0.001, df = 1, female morph: χ^2^ = 2.727, df = 2, mean morph of male partners: χ^2^ = 2.627, df = 1, all P > 0.1, N = 109; Fig 2, Table 3). Lifetime sex ratio of fathers was predicted by their own melanin morph and territory quality, but not by their LRS or longevity (Table 4). Over their lifetimes, light fathers fledged significantly higher proportion of daughters than dark and intermediate fathers (Fig 2). Males from pairs in better territories also sired more daughters. However, none of these effects remained significant when reproductive careers consisting of single broods were excluded. With increasing LRS, lifetime sex ratio approached equity (absolute difference between lifetime sex ratio and 0.5, Pearson r = -0.451, df = 94, P < 0.001).

### LRS, longevity and sex-specific chick mortality

**Fig 3:**
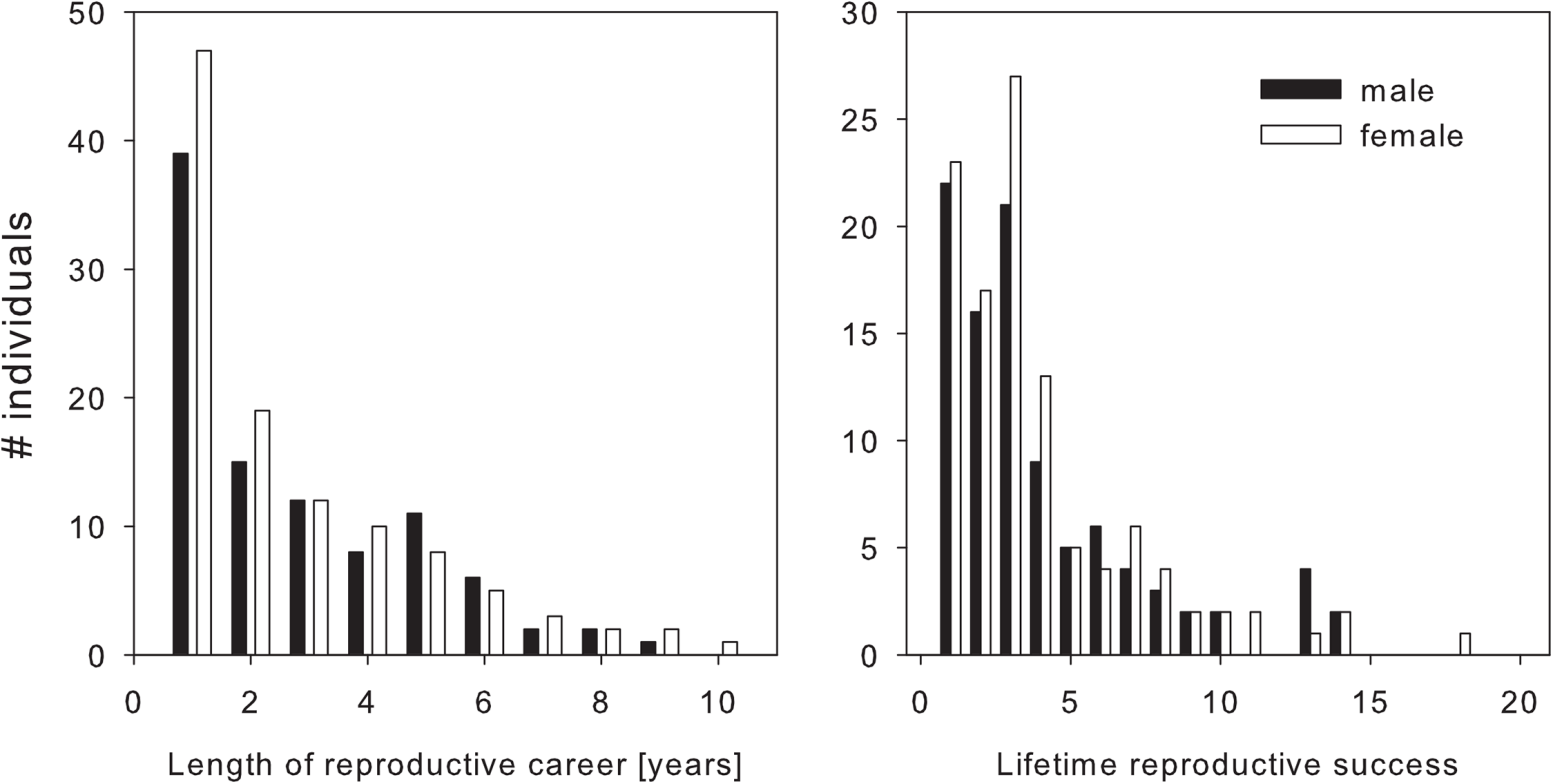
Histograms of observed length of reproductive career in years and lifetime reproductive success (LRS, total number of fledglings) of adult female and male buzzards (# individuals) with completely known LRS and lifetime sex ratio. LRS distributions of males and females were statistically indistinguishable.

There were no differences in the distributions of LRS or longevity between males and females with completely known life histories and sexed offspring (Poisson GLMs with log link, difference between sexes: LRS χ^2^ = 0.017, df = 1, P = 0.895, N = 205; longevity χ^2^ = 0.080, df = 1, P = 0.777, N = 205; Fig 3, Tables 5 and 6).

**Table 6:** Properties of distributions of lifetime reproductive success (LRS, total number of fledglings produced over the individual lifetime) and length of reproductive career of adult female and male buzzards with completely sampled offspring.

	LRS		Length of reproductive career	
	Female	Male	Female	Male
Range (min-max)	1–18	1–14	1–10	1–9
Mean (SE)	3.96 (0.312)	4 (0.339)	2.72 (0.207)	2.78 (0.207)
SD	3.257	3.325	2.161	2.027
Variance	10.610	11.053	4.688	4.110
Median	3	3	2	2
Skewness (SE)	1.815 (0.231)	1.527 (0.246)	1.369 (0.231)	1.009 (0.249)
Curtosis (SE)	3.733 (0.459)	1.806 (0.488)	1.292 (0.459)	0.182 (0.488)

Out of 43 nestlings known to have died before fledging, 16 were female and 27 were male. Nestling mortality was not significantly sex-biased, but tended to be predicted by vole score and was significantly predicted by the interaction of vole score and nestling sex (binomial GLM with logit link: vole score deviance = 5.263, df = 2, P = 0.072; nestling sex deviance = 2.571, df = 1, P = 0.109; vole score × sex deviance = 6.666, df = 2, P = 0.036, N = 1780). Years with intermediate vole score tended to have lower mortality than both high and low vole years. In high vole years, male nestlings had significantly higher mortality than females. Broods with known mortality cases were larger before but not after mortality (broods without mortality, mean size = 2.00 ± 0.03; broods before mortality, mean size = 2.63 ± 0.14, χ^2^ = 20.295, N = 884, P < 0.001; broods after mortality, mean size = 2.12 ± 0.14, χ^2^ = 0.664, P = 0.415, N = 43). Broods with mortality cases were male-biased both before (mean male fraction before mortality = 0.662 ± 0.046, N = 43; broods without mortality mean = 0.495 ± 0.0138, N = 841; χ^2^ = 7.253, P = 0.007) and after mortality (mean male fraction after mortality = 0.714 ± 0.057, N = 39; χ^2^ = 11.203, P = 0.001). Nestling mortality was not explained by territory quality or its interaction with nestling sex.

## Discussion

In common buzzards, we predicted a male-biased sex ratio under harsh conditions such as vole-poor years and in low-quality territories, corresponding to the “local adaptation hypothesis”. At the same time, we predicted more females to be produced earlier in the breeding season, corresponding to the “early sex hypothesis”. However, in common buzzards we found no systematic sex-ratio variation in response to survival-related variables such as food abundance alone or laying date. The overall scarcity of sex ratio correlations might be due to the similarity of distributions of LRS, length of reproductive career and infant mortality between sexes. The distribution of these basic fitness components was very similar between buzzard sexes. Such a pattern could reduce the scope for adaptive sex ratio adjustment.

The most consistent variable explaining buzzard nestling sex ratio was territory quality. In accordance with the “local adaptation hypothesis”, more males were produced in poor-quality territories (Fig 1). Frequently-used territories are associated with higher reproductive success and can therefore be considered to be better [40]. We are still unaware what features make a territory preferable for buzzards. However, annual food availability reflected by vole abundance showed no direct effects on offspring sex ratio. Additionally, we could not find an effect of territory quality on nestling mortality. Therefore it needs to be investigated in more detail at which developmental phase the bias in sex ratio arises.

Surprisingly, despite the absence of strong indicators for the adaptive value of biased sex ratios, we found a sex ratio pattern in relation to plumage morphs in buzzards. On the one hand, under poor food conditions, dark and light females produced more males, while intermediately-coloured females produced more females (Fig 1). The sex allocation of extremely-coloured females is in line with the “local adaptation hypothesis”. Intermediate females have higher fitness and may overall be of higher quality than extreme morphs [19]. Thus, intermediate females might be less resource-restricted and able to allocate offspring sex in a contrasting way to utilize the higher reproductive value of females in the given cohort despite harsh conditions [49]. A similar interaction between local conditions and melanin colouration has been found to affect sex allocation in barn owls [31]. Although the interaction of plumage morph and food abundance explained population-wide nestling sex, its significance should be treated cautiously since it was not part of the best explanatory models and a conservative interpretation would be that it does not contribute to explaining nestling sex.

On the other hand, morph explained male lifetime sex ratio, with light males having more female offspring over their lifetimes. Females often adapt their offspring sex ratio to features of their partners, even in monogamous species with low extra pair paternity [50]. This effect might be connected to differences between morphs and sexes in aggressiveness, parasitism and capability to cope with food limitations (see Introduction; [20, 21, 27, 51]). However, melanin morph in buzzards is inherited from both parents and it should be further investigated why offspring sex ratio would be influenced only by morph of the male partner [19, 27].

Any apparent bias of lifetime sex ratio decreased with higher offspring numbers. Too few buzzards had sufficiently high offspring numbers to identify sample-size independent extremes in lifetime sex ratio. Thus, lifetime sex ratio as a measure of allocation flexibility would be better applicable to organisms with larger litter sizes. So far, we cannot recognize individual constraints on sex allocation in buzzards.

While the “costly sex hypothesis” predicts higher female nestling mortality, especially in years of low food abundance, we found a trend for higher mortality of the smaller, supposedly less demanding males in vole-rich years. However, in such years, parents might be prone to overestimate their own provisioning capabilities and female chicks may be better able to outcompete their male siblings for prey [6, 52]. Higher mortality of males may occur also for competition-independent cryptic reasons. For example, if years with high prey abundance should coincide with years with high parasite abundance a lower immune-competence of males could lead to their higher mortality. Competition-independent mortality might explain why males died more often in male-biased and not in female-biased broods [53]. Our sample of nestling mortality was small but our result, showing overall balanced infant mortality between sexes, are supported by findings in goshawks and sparrowhawks, where also no sex-biased nestling mortality has been found [45, 54].

We did not find any patterns in LRS and longevity supporting the “early sex hypothesis” and correspondingly, we did not find any sex-ratio bias along hatching date between broods. Unfortunately, a direct test of this hypothesis by comparing the life histories of individuals of both sexes born on the same date is currently impossible for highly mobile and long-lived species such as buzzards. Nonetheless, if individuals of one sex occasionally have longer breeding careers and produce more offspring overall, the distributions of LRS and longevity should differ between the sexes. Longevity of both sexes is similar in our population but a recent study found adult survival of male buzzards to increase faster than survival of female buzzards [29]. This pattern may arise because males can profit more from increasing winter food availability under rapidly changing climate conditions. A difference in adult survival between sexes may be a significant cause for sex allocation in the future.

In summary, we did not find clear differences in LRS, length of reproductive career or mortality that may provide a basis for sex allocation in common buzzards. Only after these analyses we can comprehend why several expected life history traits and environmental conditions do not explain offspring sex ratio. However, offspring sex ratio was explained by environmental quality and its interaction with parental phenotype. This indicates potential phenotype-specific adaptation of sex allocation to local conditions and emphasizes the need for further examination of sex allocation in colour-polymorphic species.

## Supporting Information

S1 Datasets Buzzard nestling sex and lifetime sex ratio. Datasets on buzzard nestling sex, nestling mortality, lifetime sex ratio and reproductive career of adult buzzards used by Chakarov et al. “Territory quality and plumage morph predict offspring sex ratio variation in a raptor”.
(TXT)
